# Magnetically activated adhesives: towards on-demand magnetic triggering of selected polymerisation reactions†
†Electronic supplementary information (ESI) available: X-ray powder diffraction patterns, XPS, Raman and FTIR spectra, magnetisation curves, plot of conversion of monomer, tables presenting polymerisation conditions and photographs and videos demonstrating magnetically triggered adhesion of metal and glass plate combinations. See DOI: 10.1039/c7sc03474a



**DOI:** 10.1039/c7sc03474a

**Published:** 2017-09-26

**Authors:** Gemma-Louise Davies, Joseph Govan, Renata Tekoriute, Raquel Serrano-García, Hugo Nolan, David Farrell, Ory Hajatpour, Yurii K. Gun'ko

**Affiliations:** a Department of Chemistry , University College London , 20 Gordon Street , London WC1H 0AJ , UK . Email: gemma-louise.davies@ucl.ac.uk; b School of Chemistry , CRANN Institute , Trinity College Dublin , Dublin 2 , Ireland . Email: igounko@tcd.ie; c Henkel Ireland Operations & Research Limited , Tallaght Business Park, Whitestown, Tallaght , Dublin 24 , Ireland; d ITMO University , 197101 , St. Petersburg , Russia

## Abstract

On-demand initiation of chemical reactions is becoming increasingly popular in many areas. The use of a magnetic field to trigger reactions is an intriguing concept, with vast potential in both research and industrial settings, though it remains a challenge as yet unsolved. Here we report the first example of on-demand magnetic activation of a polymerisation process using an anaerobic adhesive formulation as an example of this new approach toward triggering polymerisation reactions using an external magnetic field. Our strategy involves the use of a colloidal system comprising functional methacrylate ester monomers, peroxide and Cu^II^-salt as polymerisation initiators and magnetic nanoparticles coated with an oxidising shell. This unique combination prevents reduction of the reactive transition metal (Cu^II^) ion by the metal substrates (steel or aluminium) to be joined – hence inhibiting the redox radical initiated cationic polymerisation reaction and efficiently preventing adhesion. The polymerisation and corresponding adhesion process can be triggered by removal of the functional magnetic particles using a permanent external magnet either prior to formulation application or at the joint to be adhered, enabling the polymerisation to proceed through Cu^II^-mediated reduction. This new approach enables on-demand magnetically-triggered reaction initiation and holds potential for a range of useful applications in chemistry, materials science and relevant industrial manufacturing.

## Abstract 2

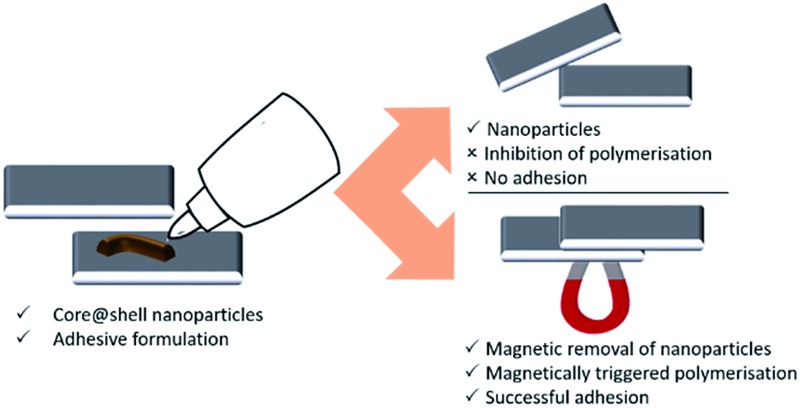
We demonstrate a new strategy to inhibit and trigger polymerisation of an adhesive formulation, utilising colloidal core@shell CoFe_2_O_4_@MnO_2_ magnetic nanoparticles.

## Introduction

On-demand triggering of reactions is a highly important area of chemistry, providing the opportunity to control chemical processes from a lab to an industrial scale. Polymerisation reactions can be successfully activated using thermal, chemical, photochemical, redox and mechanical means, showing effective modulation of reactions and resulting products.^1^ There are drawbacks to these approaches, however, including difficulties in precise controllability and delays in polymerisation initiation, though significant strides have been made towards addressing these issues in recent years.^2,3^ The activation of polymerisation reactions using a simple magnetic stimulus (*e.g.* an external magnetic field) would open up new opportunities in chemistry and materials science, providing a fast and energy efficient approach to triggering chemical reactions. Despite these distinct advantages, this technology remains a great challenge and, to the best of our knowledge, there have been no reports to date on the initiation of chemical processes by magnetic stimuli.

Anaerobic adhesives and sealants form the basis of a multi-million dollar industry and are used every day, as well as in diverse industrial applications, including the chemical bonding and locking of threaded parts, retention of cylindrical machine components and sealing of porous metal casings, amongst other important uses.^4^ Anaerobic adhesives are single component adhesives that are generally comprised of mixtures of mono- and multi-functional methacrylate ester monomers with cure chemistry based on a redox radical initiator system. At ambient temperatures and in the presence of oxygen, such anaerobic adhesives remain stable and un-reacted for extended periods of time. Normally, polymerisation is initiated when oxygen is excluded upon assembly of suitably active substrates (*i.e.* redox agents) with the adhesive.^4–7^ However, on fast-moving production lines, durable and highly reactive adhesive chemistries with short cure times and conventional cyanoacrylate based superglues are not considered appropriate or structurally robust enough for many applications.^7,8^ As the reactivity of the redox initiation used to promote rapid anaerobic adhesive polymerisation increases, a concomitant decrease in storage stability of the adhesive composition is observed, along with handling problems. Thus, highly reactive adhesive processing necessitates the development of novel means of stabilisation, control and activation to enable ease of use of the adhesive formulation. It is therefore of great importance to develop new methods for both controlling and triggering the polymerisation processes in rapid-curing anaerobic adhesives for domestic and large scale industrial applications. Photo-, pressure- and heat-activation of adhesives are very well known, though such approaches are not always convenient.^7,9,10^ Widely used photo-activated adhesives, for example, normally require high energy ultraviolet light and addition of expensive photoinitiators.^6^ Photoinitiation is also inconveniently limited to only light-transparent substrates. Pressure and heat activation are energy demanding activation techniques, which also frequently result in the damage of substrates (*e.g.* plastics) during the adhesion process.^10^ An interesting new approach involving adhesive curing through electrochemical triggering, though efficient, has slow response times (∼20 min for 10% conversion to occur);^2^ low-voltage electrochemical activation has recently been reported to solve some of these limitations,^11^ though scale-up can be challenging due to limitations associated with mass-transfer to the working electrode.

Magnetic nanoparticles are very well known and, due to the ease of their manipulation by an applied external magnetic field, have found applications in a variety of fields – from biomedicine^12,13^ to catalysis, where they have been used as convenient substrates for homogeneous catalysts, enabling immobilisation, magnetic recovery and convenient catalyst recycling.^9,14–20^ Nevertheless, the use of magnetic nanoparticles for the inhibition and/or magnetic triggering of polymerisation reaction processes has not been explored to date. In this manuscript we report an anaerobic redox radical initiated polymerisation process which can be activated on-demand using an external magnetic field: we demonstrate the magnetic triggering of an anaerobic adhesive formulation using carefully designed functional core@shell magnetic nanoparticles. Our approach of triggering chemical reactions through the application of an external magnetic field could potentially be useful in chemical reaction inhibition, control and initiation of a variety of chemical processes.

## Experimental

### General details

A JEOL JEM-2100, 200 kV LaB_6_ transmission electron microscope operated at 120 kV with a beam current of ∼65 mA was used to image nanoparticle samples. Aqueous suspensions were drop-cast onto a formvar coated copper grid for imaging. Size analysis was carried out using ImageJ software. X-ray powder diffraction was performed using a Siemens-500 X-ray diffractometer. Powder samples were adhered on silica glass using silica gel and overnight spectra were run for all samples. Diffractograms were compared to the JCPDS database. FTIR spectroscopy was performed using a Perkin Elmer Spectrum One NTS FTIR spectrometer. C=C bond monitoring experiments were conducted using a Perkin Elmer Spectrum 100 FTIR spectrometer with a diamond ATR attachment. Micro Raman spectra were recorded using a Renishaw 1000 micro-Raman system fitted with a Leica microscope and Grams Research TM analysis software. The excitation wavelength was 633 nm from a Renishaw RL633 He–Ne laser. Vibrating sample magnetometry (VSM) was carried out at room temperature with field applied up to 1 Tesla using a home-built machine. VSM was calibrated using a nickel sample of known mass; magnetisation values are representative of the total mass of the sample. X-ray photoelectron spectroscopy (XPS) was performed on an Omicron ESCA system with an EA 125 analyser and XM1000MK II monochromated Al K X-ray source (1486.7 eV). All chemicals were used as received from Sigma-Aldrich. Ultrapure (Millipore) water was collected from a Millipore system operated at 18.2 MΩ. Steel and aluminium plates for adhesion testing (Q-Panel RS-14) were purchased from Q-Lab and were cleaned thoroughly with acetone prior to use.

### Preparation of MnO_2_ nanoparticles

MnO_2_ nanoparticles were prepared by reaction of KMnO_4_ (0.5 g, 3.14 mmol) in Millipore water (250 mL) with oleic acid (5 mL, 0.016 mol) using ultrasonication (40 min), followed by stirring (2.5 h) at room temperature.^21^ The resulting dark brown precipitate was washed 3 times with ethanol using centrifugation and dried under vacuum at 80 °C.

### Preparation of CoFe_2_O_4_@MnO_2_ core@shell nanoparticles

CoFe_2_O_4_ nanoparticles were prepared by the basic co-precipitation of cobalt(ii) nitrate hexahydrate (0.58 g, 2 mmol) and iron(ii) chloride tetrahydrate (0.80 g, 4 mmol) in deoxygenated Millipore water (100 mL) using ammonium hydroxide solution (28 v/v%, to a pH of 11) under heating at 80–90 °C for 1 h.^22^ Particles were washed with Millipore water, isolated using centrifugation and dried under vacuum at room temperature.

CoFe_2_O_4_@MnO_2_ core@shell nanoparticles were produced through controlled deposition of MnO_2_ on the surface of CoFe_2_O_4_ nanoparticles. In a typical synthesis, CoFe_2_O_4_ nanoparticles (0.15 g, 0.64 mmol) were dispersed into degassed Millipore water (153 mL) in a round bottomed flask. KMnO_4_ (0.31 g, 1.95 mmol) was then added and stirred to mix. A thermometer was inserted into the flask and the suspension was heated with vigorous stirring to 80 °C. Oleic acid (3.06 mL, 9.7 mmol) was added, and the solution was stirred for 1 h at 80 °C and then allowed to stir at room temperature overnight. The resulting nanomaterials were isolated using magnetic separation, washed four times with ethanol and the isolated material was dried under vacuum.

### Preparation of adhesive formulations

Adhesive formulations were prepared by mixing a solution of triethylene glycol dimethacrylate (TRIEGMA, 10 mL, 0.037 mol), copper(ii) tetrafluoroborate hydrate (Cu(ii), 0.088 g, 0.37 mmol) and *tert*-butyl peroxybenzoate (98%, peroxide, 1 mL, 5.26 mmol) with nanoparticles (MnO_2_, CoFe_2_O_4_, CoFe_2_O_4_@MnO_2_ or control reagents) in various ratios, as described in the text and Tables S1 and S2, ESI.†

### Testing of adhesives

**Fig. 1:**
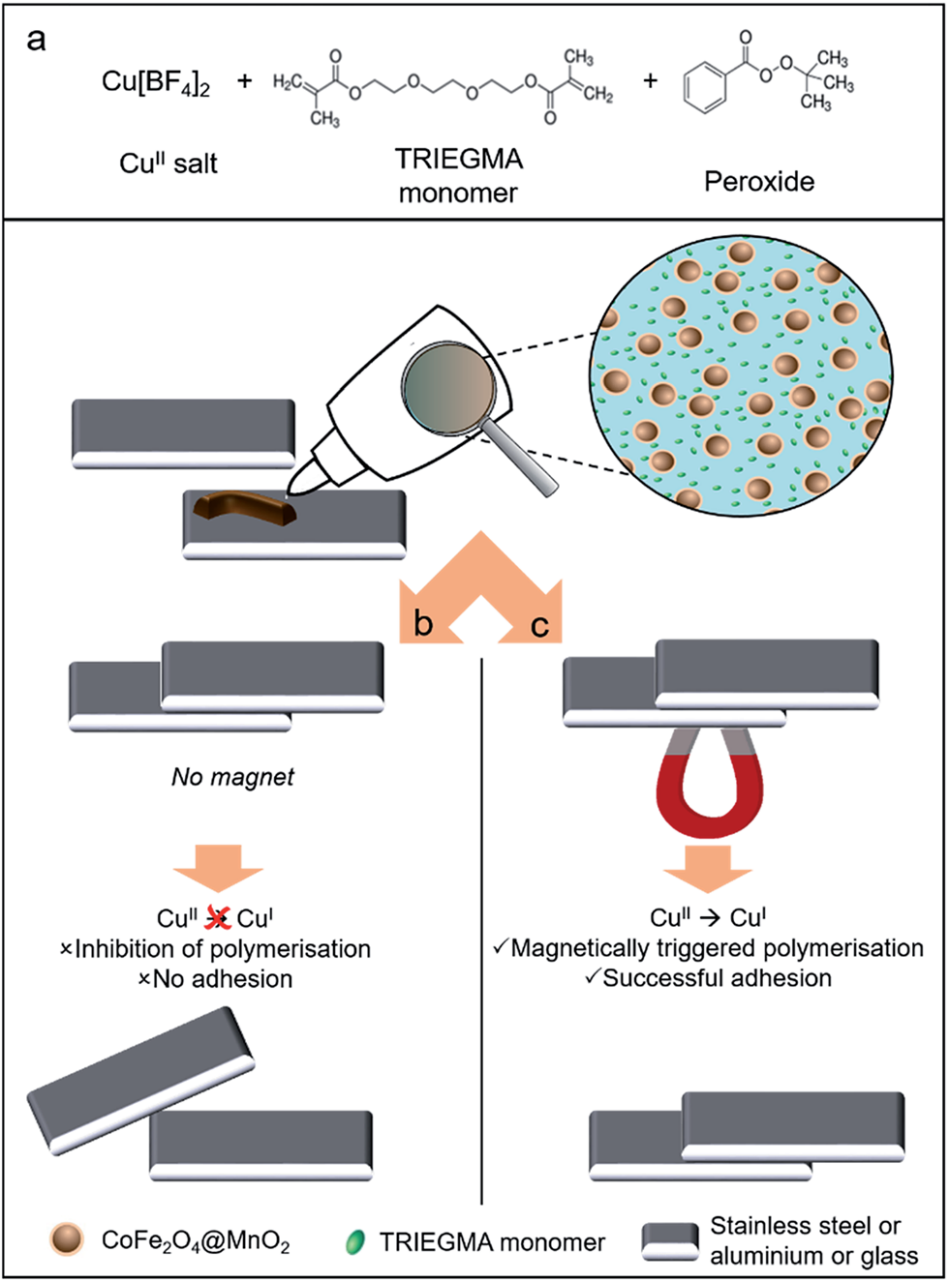
Anaerobic adhesive formulation (a). Schematic representation of core@shell magnetic nanoparticles with an oxidising outer shell (CoFe_2_O_4_@MnO_2_), which prevents reduction of a transition metal (Cu^II^) in suspension, preventing adhesion of steel/aluminium/glass plates by the anaerobic formulation in the absence of a magnetic field (b); in the presence of a magnetic field, reduction of Cu^II^ to Cu^I^ between steel/aluminium/glass plates occurs due to removal of the magnetic core@shell nanoparticles, catalysing polymerisation and resulting in successful adhesion of the plates (c).

Adhesive capability was assessed by applying the adhesive formulation (in the absence or presence of nanoparticle samples – see bullet points below) to stainless steel or aluminium test panels or stainless steel and glass test panels (see Fig. S1, ESI,† for details). The panels were held together using a clip for a set period of time (30 s) and then tested for adhesion by connecting one of the panels to a 3 kg weight and holding for 20 s. A strong adhesive joint was deemed to have been formed if the joint remained intact for a minimum of 20 s. The different formulations were applied as follows:

• As native, unaltered formulations (no nanoparticles);

• As formulations containing colloidal nanoparticles (MnO_2_ or core@shell CoFe_2_O_4_@MnO_2_ nanoparticles in varying amounts, as described in the Results and Discussion section);

• Testing of the supernatant of formulations after magnetic removal of the core@shell CoFe_2_O_4_@MnO_2_ nanoparticles (1–3 min beside a permanent magnet);

• As formulations containing colloidal nanoparticles followed by placing a magnet next to an adhesive joint between metal–metal (stainless steel or aluminium) or glass–metal (stainless steel) plate combinations (as shown in Fig. 1).

Each adhesive formulation was tested at least 5 times to confirm reproducibility, with different batches of nanoparticles.

### FTIR spectroscopy studies

Real-time FTIR spectroscopy was carried out through deposition of a droplet of the adhesive formulation either with or without nanoparticles on the diamond of the ATR system followed by attachment of a steel plate substrate. FTIR spectra were recorded every 30 seconds for 30 minutes in total. The change in the transmittance intensity of the 1637 cm^–1^ band (corresponding to C=C bond in TRIEGMA) was monitored and behaviour analysed.

The degree of vinyl monomer consumption is directly related to the increase of the IR transmittance band at 1637 cm^–1^; the % conversion of the system can be calculated from eqn (1):
1

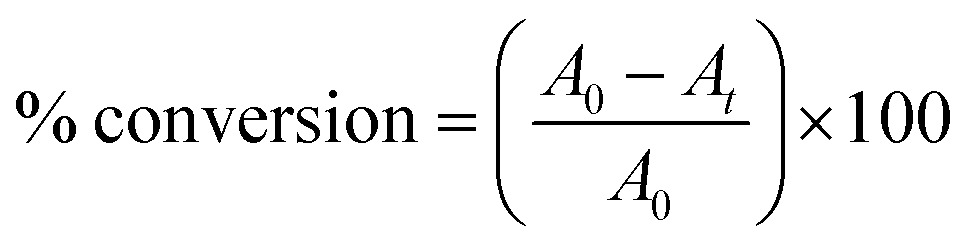

where *A*_0_ represents the absorbance at 1637 cm^–1^ at time 0 and *A*_*t*_ represents the absorbance at 1637 cm^–1^ at time *t*. Absorbance values were calculated from the transmittance data collected using eqn (2):

2 *A*_*t*_ = –*lnT*_*t*_

where *T*_*t*_ is transmittance intensity at time *t*.

### Adhesive formulation stability testing

Adhesives were tested for long-term stability using an industry-standard accelerated ageing testing protocol (Henkel Loctite STM-08, with alterations). The adhesive formulation (∼1 mL) was placed within a 12 mm diameter test-tube to which was then placed an applicator stick. Each test tube was then placed into an aluminium heating block set to 82 °C. At set times, the agglutination of each formulation (minimum 2 replications) was tested by pulling the applicator stick out of the test tube. If the adhesive formulation offered resistance to removal of the applicator stick the sample was determined to have polymerised (gelled). The adhesive formulation was tested in the absence (native formulation) and presence of colloidal nanoparticles, as described in the Results and Discussion section.

### Magnetic activation at a joint

The complete colloidal adhesive formulation (containing optimised concentration of CoFe_2_O_4_@MnO_2_ nanoparticles, TRIEGMA, copper(ii) tetrafluoroborate hydrate and *tert*-butyl peroxybenzoate, as described in ‘Preparation of adhesive formulations’) was applied to either a glass or aluminium plate. In the absence of a magnetic field, a second plate (stainless steel or aluminium, respectively) was placed on top and held in place by a clip for 30 s. After this time, the clip was removed and the plates then tested for adhesion by connecting one of the panels to a 3 kg weight and holding for 20 s. A strong adhesive joint was deemed to have been formed if the joint remained intact for a minimum of 20 s. Magnetic activation was assessed by carrying out the same procedure, but this time placing the clip-held joint beside a permanent magnet (∼0.5 T) for 30 s. The same adhesion test was repeated.

## Results and discussion

The adhesive process examined herein involves the industrially-relevant polymerisation of triethylene glycol dimethacrylate (TRIEGMA) by Cu^I^ in anaerobic conditions.^23^ Using copper(ii) tetrafluoroborate and *tert*-butyl peroxybenzoate (peroxide) as initiators of polymerisation and steel or aluminium plates, or glass and steel plates, as the substrates to be glued together, reduction of Cu^II^ to Cu^I^ occurs, initiating polymerisation of the TRIEGMA within 30 seconds and forming a strong bond between the plates.^24^ Copper-mediated polymerisation is one of the most popular routes to well-defined polymers, however only a limited number of stimuli have been shown to facilitate *in situ* triggering of such polymerisations, including quite expensive photo-irradiation (*e.g.* to produce reactive manganese radicals) and electrochemical (*e.g.* to trigger reduction of Cu^II^) approaches.^1^ In this work, we aimed to use colloidal magnetic nanoparticles (CoFe_2_O_4_) coated with a functional oxide (MnO_2_) shell which is capable of preventing Cu^II^ reduction, hence inhibiting polymerisation and adhesion. Efficient removal of the core@shell CoFe_2_O_4_@MnO_2_ magnetic particles using a permanent external magnet then enables the polymerisation process to proceed as normal – providing magnetically-triggered on-demand gluing (Fig. 1).^25^

**Fig. 2:**
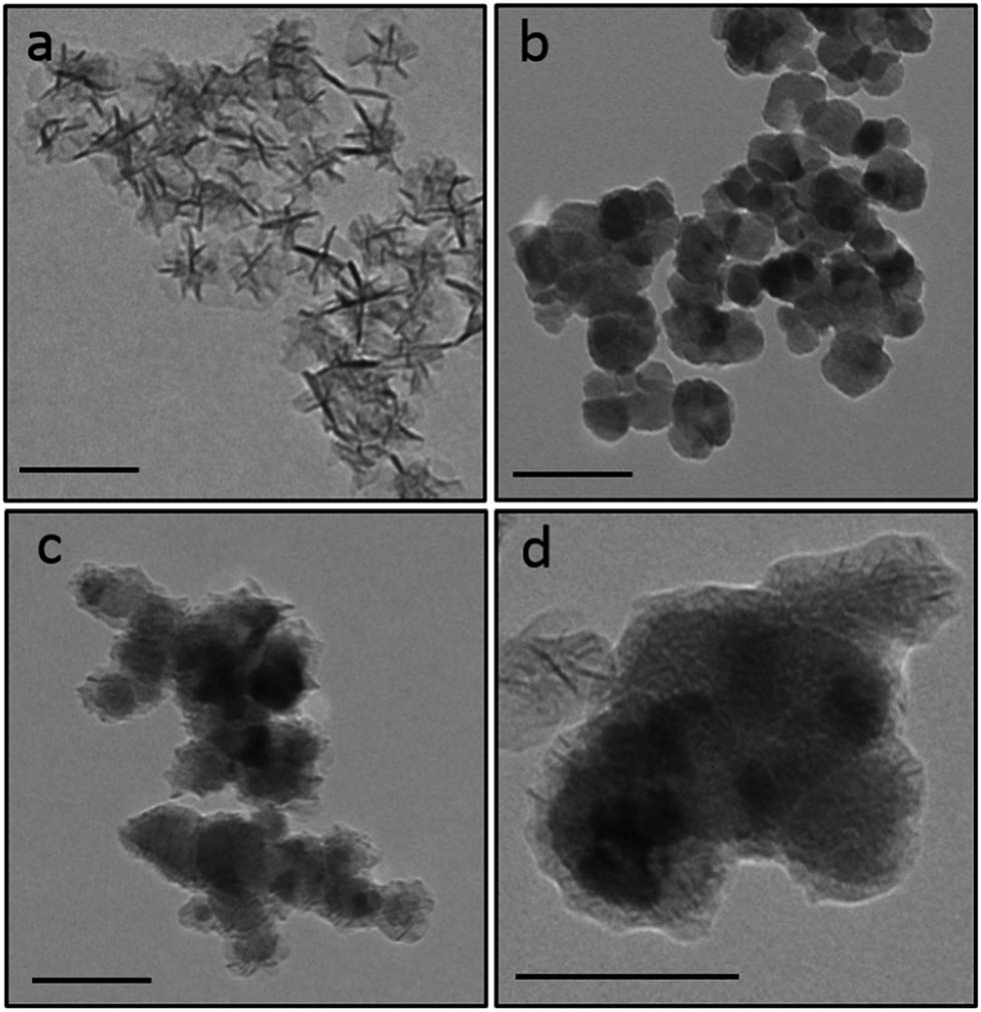
Transmission electron microscope (TEM) images of (a) MnO_2_ nanoparticles (46.7 ± 8.1 nm), (b) CoFe_2_O_4_ nanoparticles (41.0 ± 15.1 nm), (c) and (d) CoFe_2_O_4_@MnO_2_ core@shell nanoparticles (52.8 ± 19.6 nm). Scale bar is 100 nm.

Manganese dioxide (MnO_2_) is a strong oxidant (*E*° = +1.23 V *vs.* NHE) which has the potential to prevent the reduction of Cu^II^ to Cu^I^ (+0.15 V *vs.* NHE) in aqueous solutions. However, its activity in colloidal organic systems is unclear. Therefore, we initially prepared colloidal MnO_2_ nanoparticles, following published procedures,^21^ in order to test their oxidant activity by monitoring polymerisation of the adhesive formulation described above in their presence (according to Fig. S1, ESI†). Transmission electron microscopy (TEM) demonstrated the formation of star-like oleic acid stabilised MnO_2_ nanostructures (46.7 ± 8.1 nm, Fig. 2a). X-ray diffraction (XRD) confirmed their δ-MnO_2_ (birnessite-type) phase (Fig. S2, ESI†).^26,27^ A standard industrial test developed by Henkel was used for the assessment of successful anaerobic adhesion (see Fig. 1, Experimental details and Fig. S1, ESI†). The amount of MnO_2_ nanoparticles added to the adhesive formulation was initially varied to determine the optimal amount required to prevent polymerisation and hence plate adhesion (see Table S1, ESI†). It was found that a mass ratio of 2 : 1 of MnO_2_ : Cu^II^ salt or higher is necessary to successfully inhibit polymerisation. The high colloidal stability of the MnO_2_ nanoparticles in TRIEGMA and their lack of magnetic response due to their non-magnetic characteristics prevented their removal using an external magnetic field.

Cobalt ferrite (CoFe_2_O_4_) nanoparticles are a class of iron oxides which often possess high magnetic moments and saturation magnetisation values.^22,28,29^ Due to their strong and immediate attraction to external magnetic fields, CoFe_2_O_4_ nanoparticles were employed herein as a magnetic core material, which was coated with a MnO_2_ shell to create the desired magnetic and oxidising nanocomposites. Magnetic CoFe_2_O_4_ nanoparticles were initially prepared using traditional co-precipitation techniques.^30,31^ Core@shell CoFe_2_O_4_@MnO_2_ nanostructures were then produced by the reaction of pre-prepared CoFe_2_O_4_ nanoparticles with KMnO_4_ in the presence of oleic acid at 80 °C at a 3 : 1 molar ratio of KMnO_4_ : CoFe_2_O_4_. The resulting nanomaterials were washed with ethanol and isolated using magnetic separation, ensuring only coated CoFe_2_O_4_@MnO_2_ particles were retained. TEM images of the uncoated CoFe_2_O_4_ nanoparticles (41.0 ± 15.1 nm diameter, Fig. 2b) and core@shell nanostructures (52.8 ± 19.6 nm in diameter, Fig. 2c and d) showed particles with a MnO_2_ shell of 7 ± 3 nm thickness. XRD confirmed the presence of both cubic inverse spinel CoFe_2_O_4_ and δ-MnO_2_ phases (Fig. S2, ESI†). The presence of both of these oxide phases in the nanostructures was also confirmed by Raman (Fig. S3, ESI†) and FTIR (Fig. S4, ESI†) spectroscopy. Magnetisation measurements demonstrated strong saturation magnetisation values for CoFe_2_O_4_ nanoparticles (*M*_s_ = 54.2 emu g^–1^, comparable with literature particles prepared by a similar route,^22^ Fig. S5, ESI†) and CoFe_2_O_4_@MnO_2_ particles (*M*_s_ = 38.2 emu g^–1^, Fig. S5, ESI†), indicating that the core@shell nanostructures retained strong magnetisation behaviour, despite the presence of the non-magnetic MnO_2_ shell, indicating their ability to be manipulated with ease using an external magnet.

**Fig. 3:**
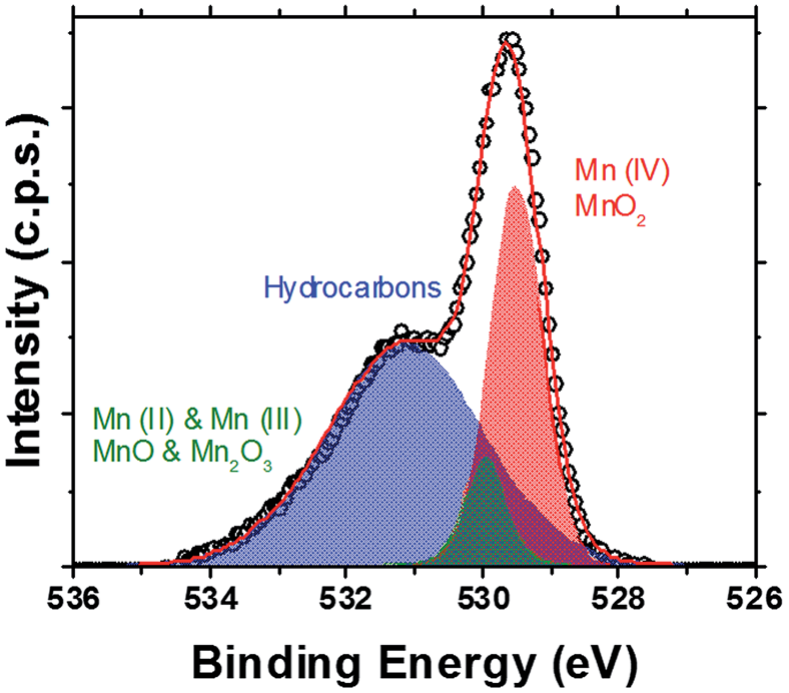
XPS (high resolution scan of the peak seen in the complete survey scan, Fig. S6, ESI†) of the O 1s core level of CoFe_2_O_4_@MnO_2_ core@shell nanoparticles. Peaks represent Mn(iv)O_2_ (red), Mn(ii)O and Mn(iii)_2_O_3_ (green) present on the nanoparticle surfaces, as well as hydrocarbons (blue) attributed to the presence of oleic acid stabilising agents used during nanoparticle preparation.

XPS studies (Fig. 3 and S6 and S7, ESI†) have shown large peaks representative of oxygen and manganese on the surfaces of the core–shell nanoparticles. Peaks representing Co and Fe indicative of CoFe_2_O_4_ core particles are present, but are low in intensity (Fig. S6, S8 and S9, ESI†). Fig. 3 demonstrates the presence of a sharp peak, centred at 529.9 eV, corresponding to O in MnO_2_ (≈80%), with a small amount of signal likely due to the presence of MnO and Mn_2_O_3_ (peak centred at 531.5 eV).^32–34^ The binding energy positions of MnO and Mn_2_O_3_ are too close together to allow feasible separation of their contributions, due to the resolution of the system used herein. Peaks representing various hydrocarbons attributed to the oleic acid stabiliser have also been observed (as labelled, Fig. 3).

The adhesion inhibition capability of these core@shell CoFe_2_O_4_@MnO_2_ nanoparticles was tested in the standard TRIEGMA based adhesive formulation (see Fig. 1, S1, ESI† and Experimental). The mass ratio of core@shell particles : Cu^II^ salt was varied to determine the optimal amount required to prevent adhesion in suspension, but subsequently allow the polymerisation to proceed upon magnetic removal of core@shell nanoparticles (Table S1, ESI†). It was found that the core@shell nanoparticles successfully inhibited polymerisation of adhesive formulations and substrate fixing at mass ratios of 3.5 : 1 of CoFe_2_O_4_@MnO_2_ : Cu^II^ and above, whereas lower ratios were not sufficient to deactivate the polymerisation process and resulted in adhesion of metal substrates in the presence of the nanocomposites. In all samples, the magnetic core@shell nanostructures could be easily removed from the adhesive formulation prior to application to joints to be fixed, using an external permanent magnet (∼0.5 T) within 1–3 minutes. For the mass ratio of 3.5 : 1 of CoFe_2_O_4_@MnO_2_ : Cu^II^, the remaining adhesive formulation (after magnetic removal of particles) then successfully fixed metal substrates within 30 s. Higher ratios of CoFe_2_O_4_@MnO_2_ : Cu^II^ (>3.5 : 1) on the other hand, did not demonstrate adhesive properties after particle removal. This may be due to the high quantities of particles used in the samples and their incomplete removal during 3 min exposure to a magnetic field. The optimised adhesive formulation (containing 3.5 : 1 CoFe_2_O_4_@MnO_2_ : Cu^II^) was left standing (at ambient temperature conditions) for three weeks. After this time, the colloidal mixture was still very stable and the presence of core–shell nanoparticles still successfully deactivated polymerisation and substrate adhesion and, upon magnetic removal of the nanoparticles, provided a formulation which was capable of triggering the polymerisation reaction and adhesion of metal substrates. This clearly demonstrates the long-term stability and activity of this novel colloidal system capable of non-permanent deactivation of polymerisation and magnetically-triggered adhesion. In comparison, the native adhesive formulation (without added stabilising nanoparticles) gelled within 24 h of storage in ambient conditions, rendering it unusable as an active adhesive.

In order to ensure that none of the individual nanocomposite components caused the deactivation/activation behaviour observed, a number of control experiments were performed (Table S2, ESI†). Oleic acid, KMnO_4_, and non-coated CoFe_2_O_4_ nanoparticles were tested in the adhesive formulation and showed no deactivation of adhesive capability in their presence and no capability of magnetically triggered adhesion.

**Fig. 4:**
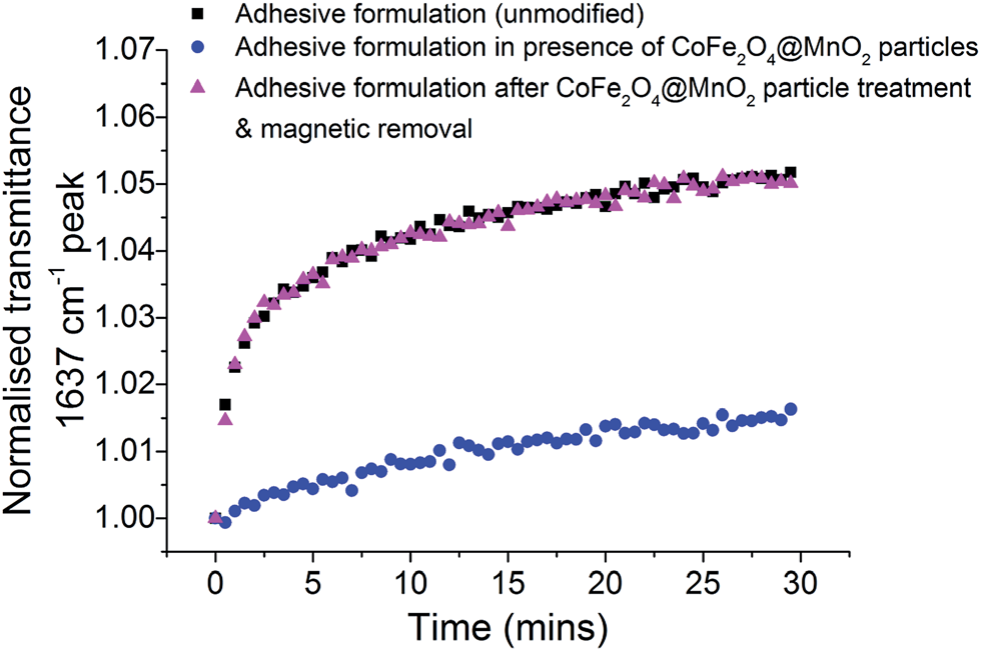
Plot of normalised transmittance of the C=C bond of TRIEGMA (1637 cm^–1^) indicative of the polymerisation of TRIEGMA in the adhesive formulation in contact with a steel substrate with respect to time. Samples measured: unmodified adhesive formulation (black), formulation with optimised CoFe_2_O_4_@MnO_2_ nanoparticles present (blue) and formulation after they had been magnetically removed (magenta).

Real-time FTIR spectroscopy can be used to analyse the progression of polymerisation of adhesive formulations, through monitoring of the C=C stretch (band maximum at 1637 cm^–1^), whose increase in transmittance intensity is characteristic of vinyl polymerisation on metal and glass surfaces.^31^ Herein, the standard unmodified glue formulation, the formulation with optimised CoFe_2_O_4_@MnO_2_ nanoparticles and the formulation after the core@shell particles had been magnetically removed were placed on an ATR FTIR system and a steel plate substrate was attached. FTIR transmittance spectra were recorded every 30 seconds for 30 minutes in total (Fig. S10, ESI†). The transmittance intensity of the 1637 cm^–1^ band of the unmodified adhesive formulation showed an initial rapid increase as C=C bonds were converted to C–C bonds during polymerisation, which then slowed over time (Fig. 4). This behaviour is typical for a rapid polymerisation process which reduces in rate as available monomer species are consumed during the fast curing process. The formulation containing optimised CoFe_2_O_4_@MnO_2_ nanoparticles showed similar behaviour, but with considerably less overall change in transmittance, indicating that only very limited polymerisation occurred under bonding conditions and the lack of significant changes in transmittance profile are indicative of remaining C=C bonds in the unpolymerised TRIEGMA monomer. Most importantly, after magnetic removal of the core@shell nanoparticles from the adhesive formulation, the trend in transmittance intensity was almost identical to that of the unmodified formulation. The restoration of the polymerisation behaviour to that of the initial unmodified adhesive formulation clearly demonstrates that the polymerisation behaviour is not negatively affected by the inclusion of the particles and remains equivalent to original, unmodified samples after nanoparticle removal.

Analysis of the conversion of the monomer with respect to curing time (Fig. S11, ESI†) supports this, with the unmodified formulation showing 17.9% conversion after 30 min cure time. In the presence of the core@shell nanoparticles on the other hand, conversion only reaches 8.0%. This degree of conversion is not enough for a strong adhesive bond to be formed, as demonstrated in previous adhesion tests (Tables S1 and S2, ESI†). After magnetic removal of the particles, the formulation reaches a 17.2% conversion after 30 min cure time, similar to the unmodified formulation, demonstrating that the formulation can still be applied as an efficient adhesive, forming strong bonds (confirmed in Tables S1 and S2, ESI†).

**Fig. 5:**
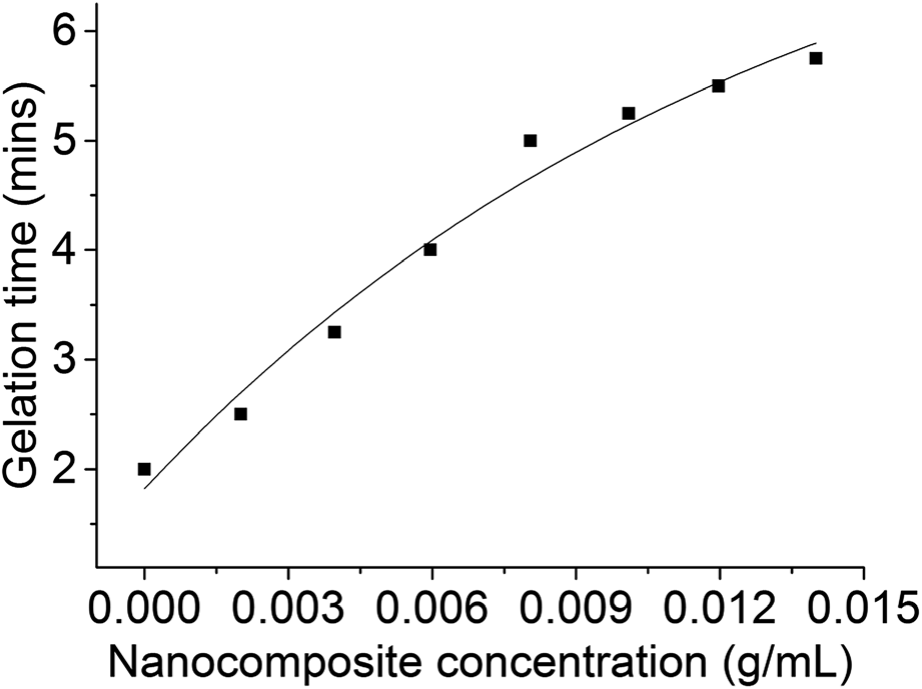
Change in gelation time with increasing concentration of CoFe_2_O_4_@MnO_2_ nanocomposites in adhesive formulation at 82 °C.

The stability of adhesive formulations (measured by assessment of gelation time) with increasing concentration of nanocomposites at 82 °C (accelerated stability testing) was performed according to a standard industry protocol (see Experimental). As expected, the stability of the active formulation increased with increasing concentration of CoFe_2_O_4_@MnO_2_ nanoparticles (Fig. 5).

**Fig. 6:**
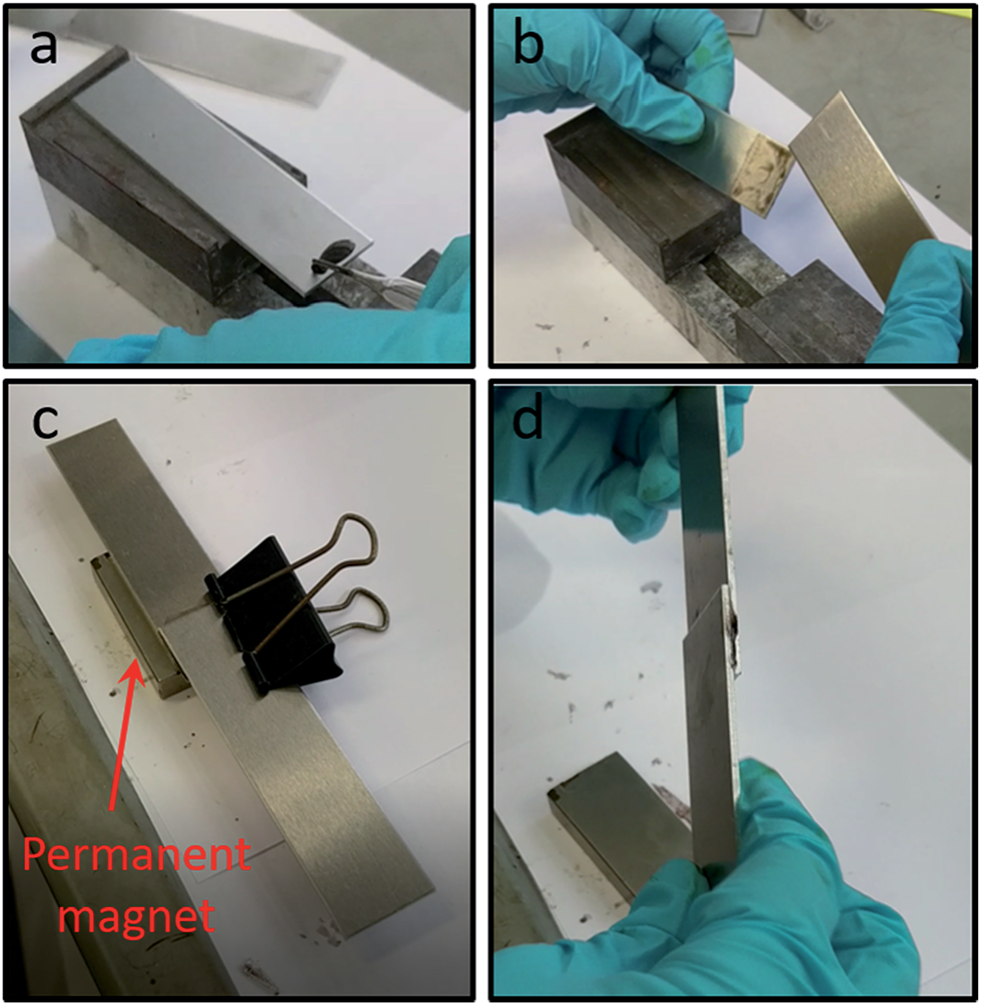
Still images showing aluminium–aluminium plate combination adhesive tests: (a) application of complete colloidal adhesive formulation containing CoFe_2_O_4_@MnO_2_ particles to a plate; (b) lack of plate adhesion in absence of a magnetic field; (c) application of a permanent magnetic field (magnet indicated by arrow) to the joint with complete colloidal adhesive formulation applied, as per (a); (d) successful plate adhesion as a result of magnet application to the joint for 30 s. Video files of lack of adhesion in the absence of a magnetic field (Video S1, ESI†) and adhesion in the presence of a magnetic field (Video S2, ESI†) to complement these images can be viewed in the ESI.†

Having demonstrated the efficacy of our nanocomposite system to prevent polymerisation and subsequently trigger it upon magnetic removal of the nanoparticles from the formulation prior to application, we next sought to demonstrate that a joint, to which our complete colloidal formulation has been applied, will only adhere in the presence of a magnetic field. We have therefore carried out experiments on combinations of aluminium–aluminium and steel–glass plate joints, to demonstrate the versatility of this approach on different substrates. Video (Video S1, ESI†) and images (Fig. 6a and b and S12a and b, ESI†) show that no adhesion between plates occurs upon application of our formulation in the absence of a magnetic field (as confirmed in prior experiments). However, if a magnet is placed alongside a joint with our optimised formulation applied, a strong adhesive joint is formed within a short (30 s) timeframe (Video S2, ESI, Fig. 6c and d and S12c and d, ESI†). This clearly demonstrates the effectiveness of our nanocomposite formulation in preventing adhesion, with successful adhesion taking place ‘on-demand’ only when triggered by the application of a magnet near the joint. This effectively removes the stabilising nanoparticles away from the formulation, destabilising it and enabling polymerisation, and hence adhesion, to take place.^25^

## Conclusions

On-demand triggered polymerisation *via* the application of an external stimulus is an increasingly important area of research, due to its enormous potential in many applications (both research and industrial).^10,11^ Recent investigations have demonstrated novel electrochemical routes to triggered polymerisation and adhesive curing, however challenges surrounding scale-up and delayed initiation of polymerisation (∼20 min after application of the stimulus) have plagued the further development of this important area.^8,9^ Herein, we have demonstrated a new strategy to inhibit and trigger the polymerisation of a known adhesive formulation, utilising colloidal core@shell CoFe_2_O_4_@MnO_2_ magnetic nanoparticles. The oxidising MnO_2_ shell efficiently prevents the reduction of a copper salt from Cu^II^ to Cu^I^, hence inhibiting the redox radical initiated cationic polymerisation of an ethyleneglycol dimethacrylate (TRIEGMA) monomer, effectively preventing the adhesion of metal–metal and metal–glass joint combinations. On-demand activation of the polymerisation process can be prompted through the application of a magnetic field, which quickly removes these stabilising nanoparticles through magnetic attraction. We have demonstrated the efficient and reliable nature of our system through carrying out direct magnetic application tests, wherein an applied formulation containing the stabilising colloidal particles results in the on-demand formation of a strong adhesive joint only upon application of a magnetic field to the joint to be fixed.^25^

## Conflicts of interest

There are no conflicts to declare.
