# Supplementary material for: Magnetically activated adhesives: towards on-demand magnetic triggering of selected polymerisation reactions
Source: Chem Sci. 2017 Sep 26;8(11):7758–64. doi: 10.1039/c7sc03474a (PMC5674535; doi:10.1039/c7sc03474a)
Supplement: Supplementary file 1 [file SC-008-C7SC03474A-s001.pdf]

Electronic Supplementary Material (ESI) for Chemical Science.
This journal is © The Royal Society of Chemistry 2017

Magnetically activated adhesives: towards on-demand magnetic triggering of selected polymerisation reactions

Gemma-Louise Davies,[a*] Joseph Govan,[b] Renata Tekoriute,[b] Raquel Serrano-García,[b] Hugo Nolan,[b] David Farrell,[c] Ory Hajatpour[c] and Yurii K. Gun'ko[b,d*]

[a] Department of Chemistry, University College London, 20 Gordon Street, London WC1H 0AJ, UK, [b] School of Chemistry and CRANN Institute, Trinity College Dublin, Dublin 2, Ireland, [c] Henkel Ireland Operations & Research Limited, Tallaght Business Park, Whitestown, Tallaght, Dublin 24, Ireland and [d] ITMO University, 197101, St. Petersburg, Russia.

Electronic Supplementary information

Datasets are available open access (DOI: 10.14324/000.ds.1575710), and can be found by following the link: dx.doi.org/10.14324/000.ds.1575710.

Figures

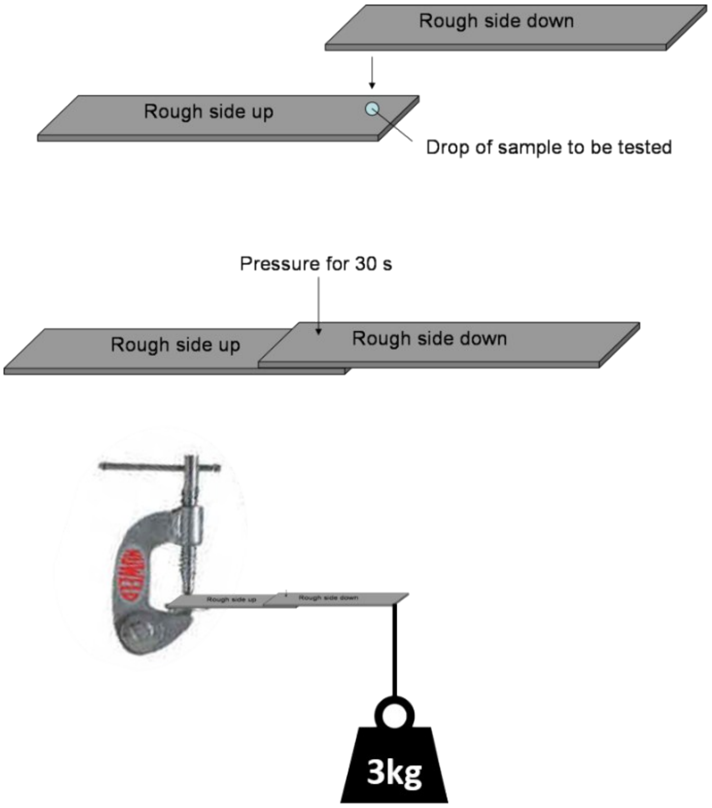

Figure S1. General scheme for adhesion procedure and testing of anaerobic adhesive formulations on plates, which can be stainless steel, aluminium or a combination of steel/aluminium and glass.

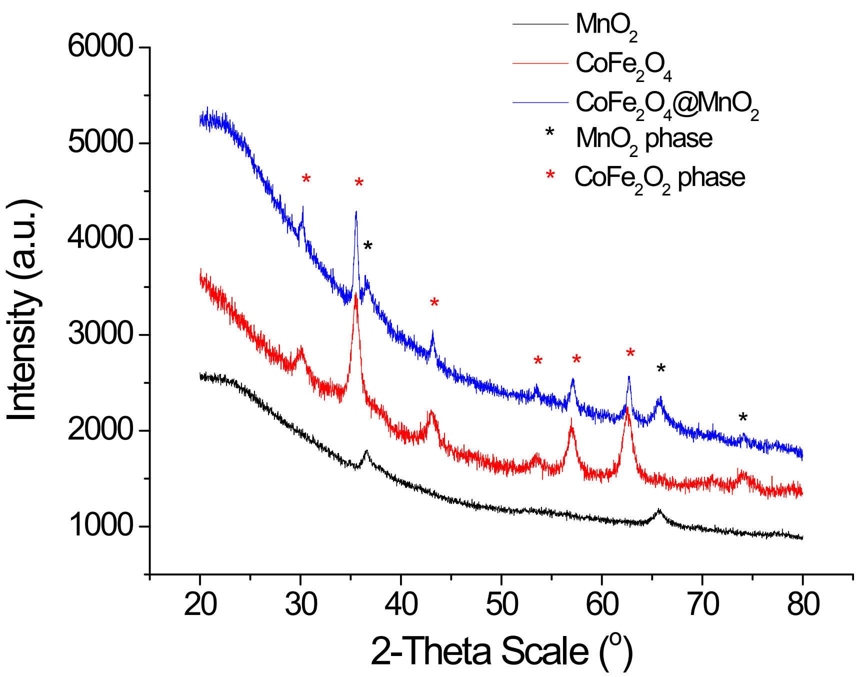

Figure S2. X-ray powder diffraction patterns of MnO_2 (black), $CoFe_2O_4$ (red) and $CoFe_2O_4$@MnO_2 (blue) nanocomposites, stars represent the δ-MnO_2 phase (black) and $CoFe_2O_4$ (red) cubic spinel phase present in the core@shell particles, as labelled.

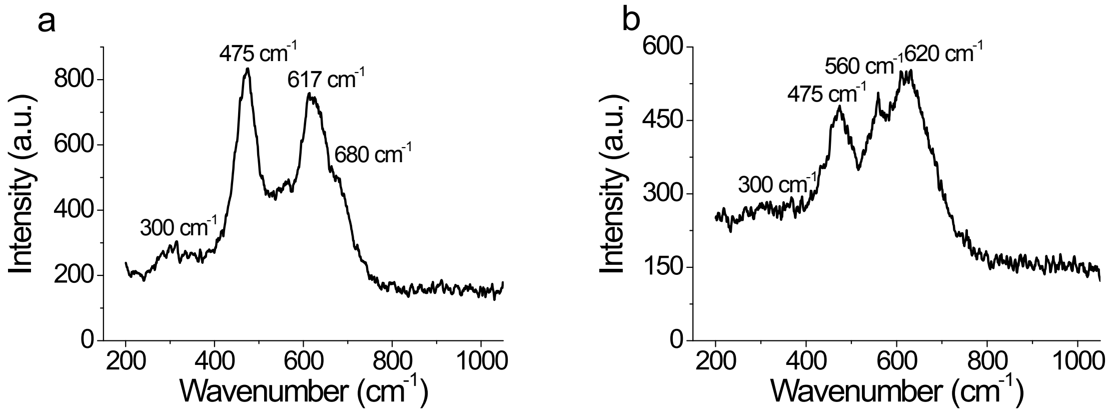

Figure S3. Raman spectra of a) $CoFe_2O_4$ nanoparticles and b) $CoFe_2O_4$@MnO_2 core@shell nanoparticles. Raman modes at 680 cm^{-1}, 617 cm^{-1}, 475 cm^{-1} and 300 cm^{-1} are characteristic of the cubic inverse spinel structure of $CoFe_2O_3$;[1] peaks at 560 cm^{-1} and 620 cm^{-1} (increased intensity compared to $CoFe_2O_4$ alone) are characteristic of δ-MnO_2.[2]

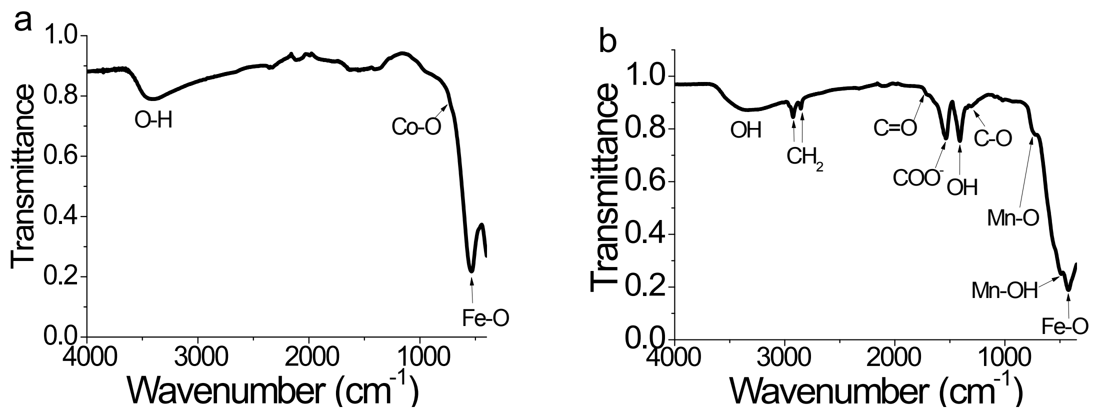

Figure S4. FTIR spectra of a) $CoFe_2O_4$ nanoparticles showing Fe-O stretching vibrations (530 cm^{-1}), a small shoulder representing Co-O stretching vibrations (720 cm^{-1}) and hydroxyl group stretching vibrations (3400 cm^{-1}), as labelled and b) $CoFe_2O_4$@MnO_2 nanocomposites showing Mn-O stretching and Mn-O-H bending vibrations (725 and 490 cm^{-1}), characteristic Fe-O vibrations, hydroxyl groups stretches (labelled, as in (a)) as well as distinctive peaks for oleic acid, including asymmetric and symmetric CH_2 stretches (2925 and 2850 cm^{-1}), C=O and C-O stretches (1712 and 1340 cm^{-1}), O-H (1410 cm^{-1}) and asymmetric COO^- stretches (1535 cm^{-1}), as labelled.[2, 3]

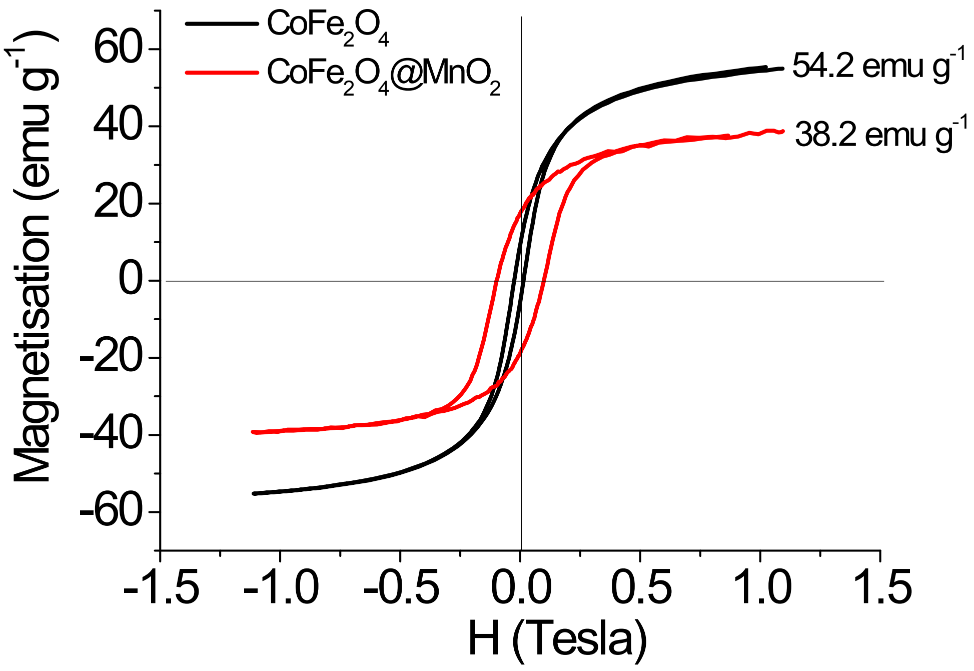

Figure S5. Magnetisation curves of $CoFe_2O_4$ and $CoFe_2O_4$@MnO_2 nanocomposites. Samples demonstrate hysteresis loops indicative of ferromagnetism. Saturation magnetisation values (as labelled, based on total mass of sample) decrease upon MnO_2 coating, as expected due to the presence of the non-magnetic MnO_2 layer.

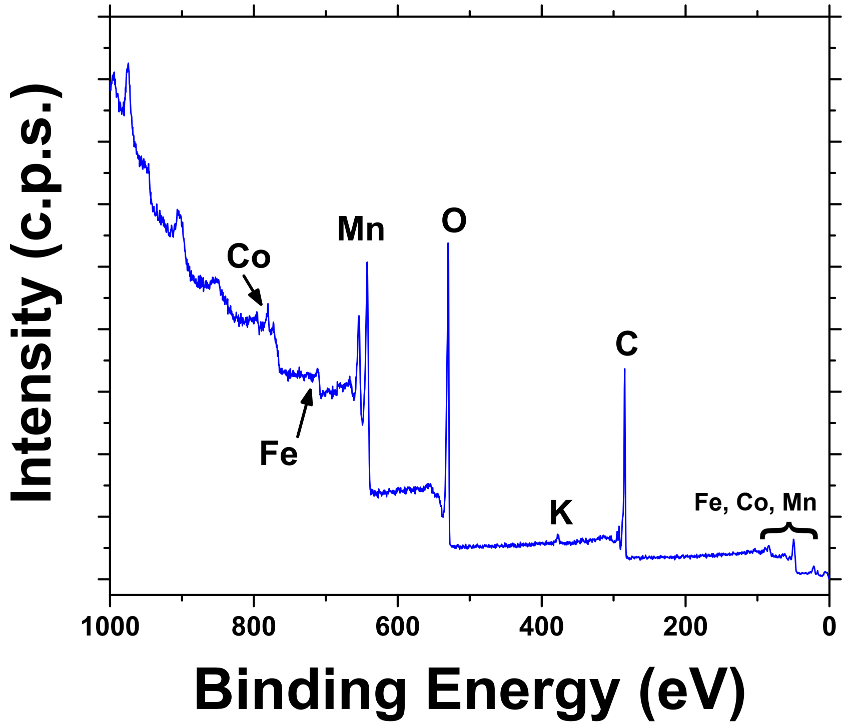

Figure S6. Complete XPS spectrum of $CoFe_2O_4@MnO_2$ core@shell nanoparticles with elements labelled.

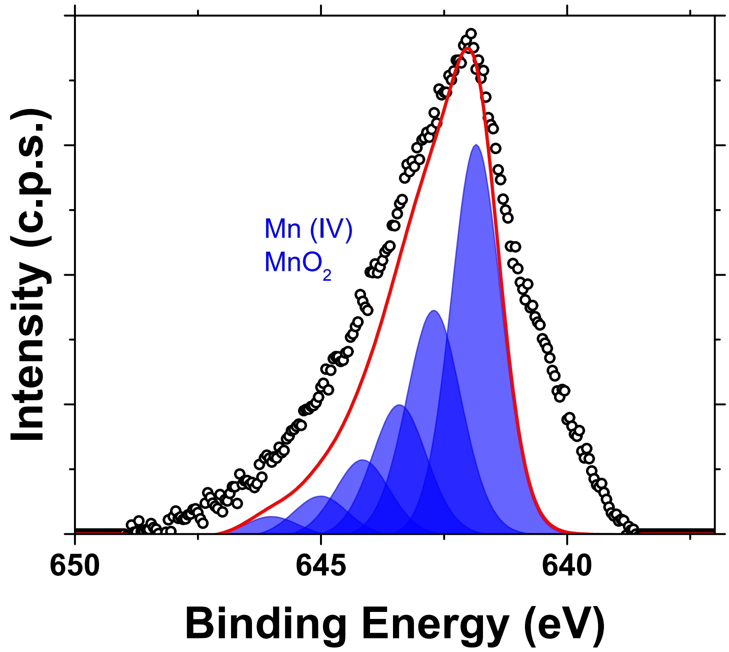

Figure S7. Mn 2p core level XPS spectrum of $CoFe_2O_4@MnO_2$ core@shell nanoparticles.

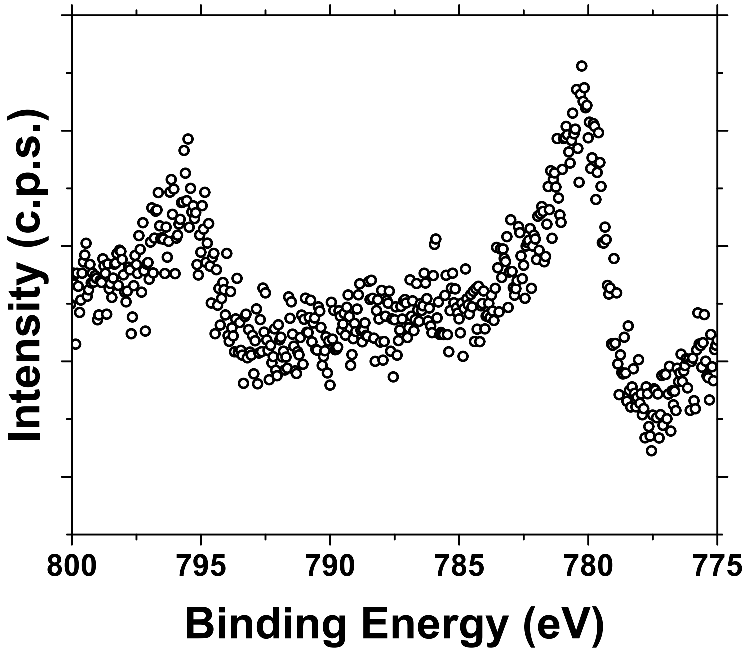

Figure S8. Co 2p core level XPS spectrum of $CoFe_2O_4$@MnO_2 core@shell nanoparticles.

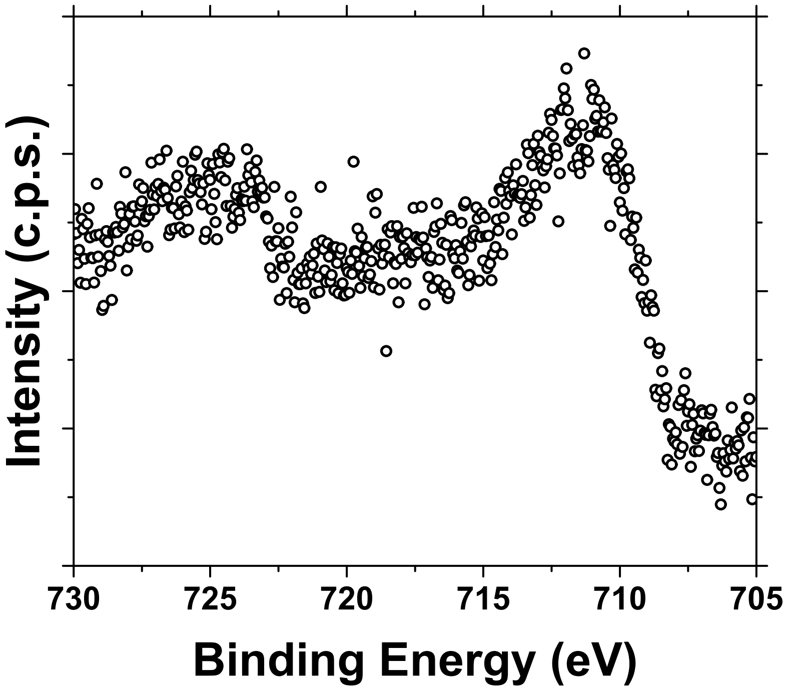

Figure S9. Fe 2p core level XPS spectrum of $CoFe_2O_4$@MnO_2 core@shell nanoparticles.

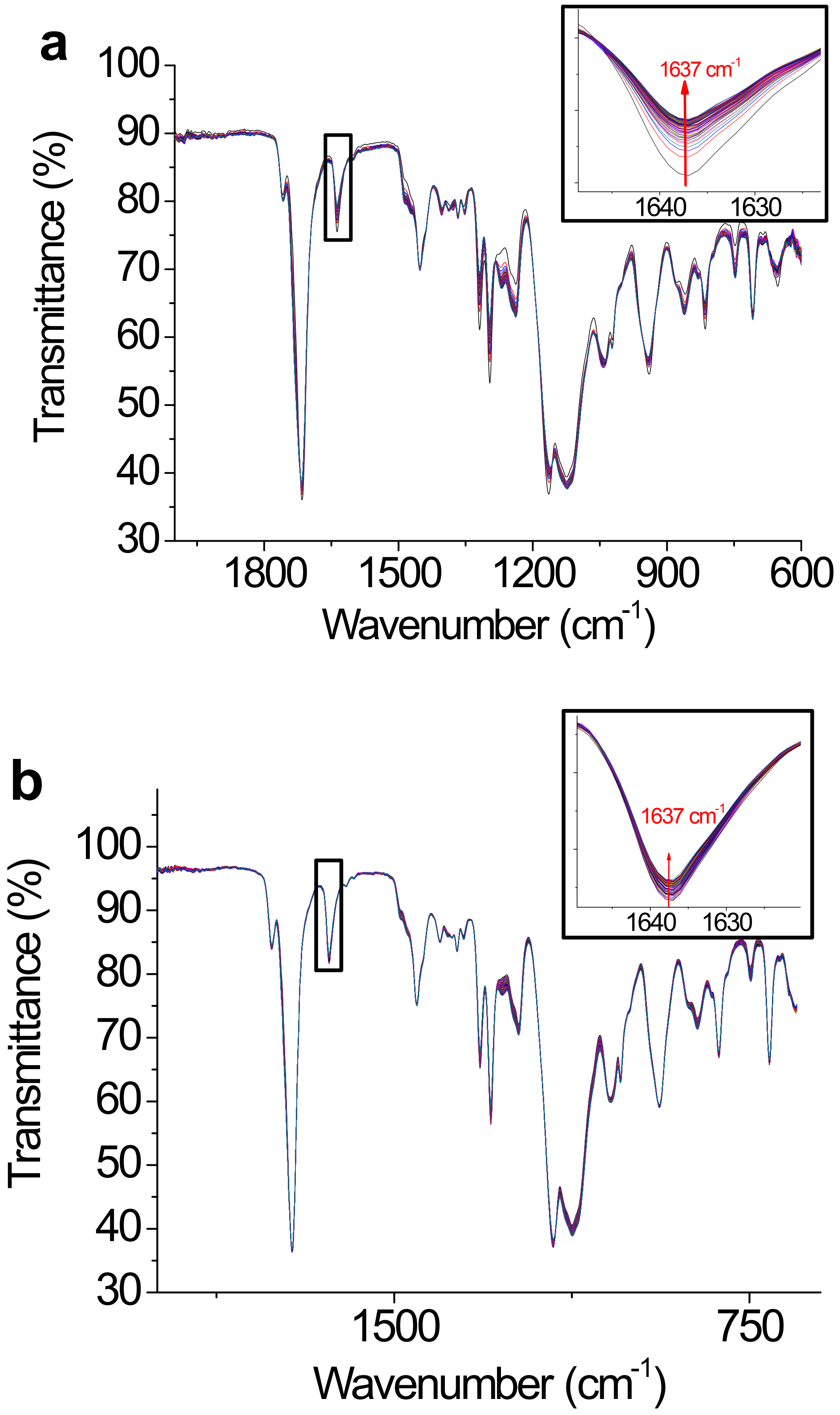
a
Transmittance (%)
Wavenumber (cm-1)
1637 cm-1
b
Transmittance (%)
Wavenumber (cm-1)
1637 cm-1

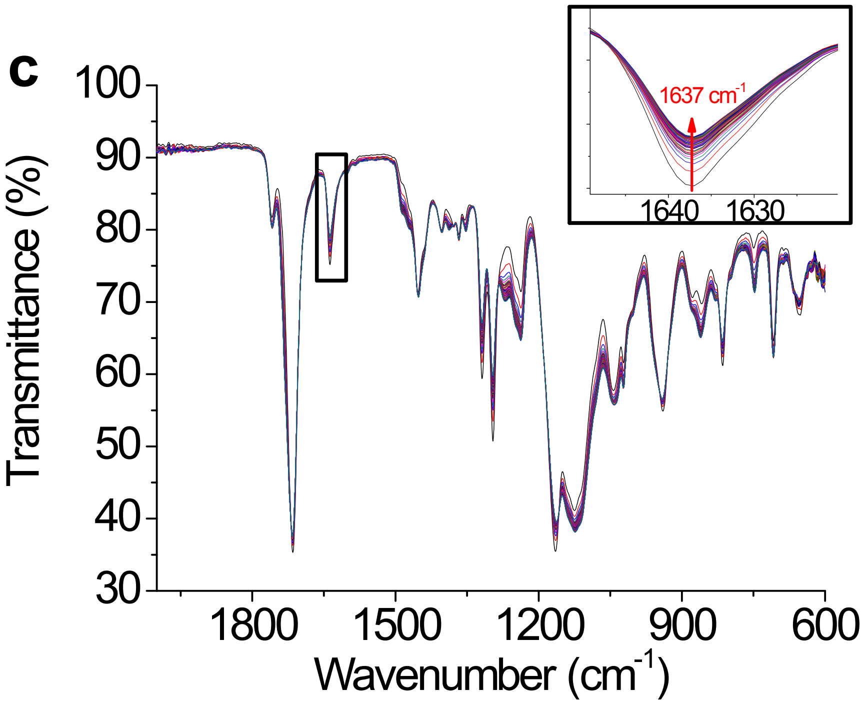

Figure S10. a) FTIR transmittance spectra of unmodified adhesive formulation monitored over time. Inset shows increase of band at 1637 cm^{-1} indicative of reduction in concentration of C=C bond over time. b) FTIR transmittance spectra of adhesive formulation in the presence of optimised ratio of $CoFe_2O_4$@MnO_2 nanoparticles monitored over time. Inset shows increase of band at 1637 cm^{-1} indicative of reduction in concentration of C=C bond over time. c) FTIR transmittance spectra of adhesive formulation after magnetic removal of $CoFe_2O_4$@MnO_2 nanoparticles monitored over time. Inset shows increase of band at 1637 cm^{-1} indicative of reduction in concentration of C=C bond over time.

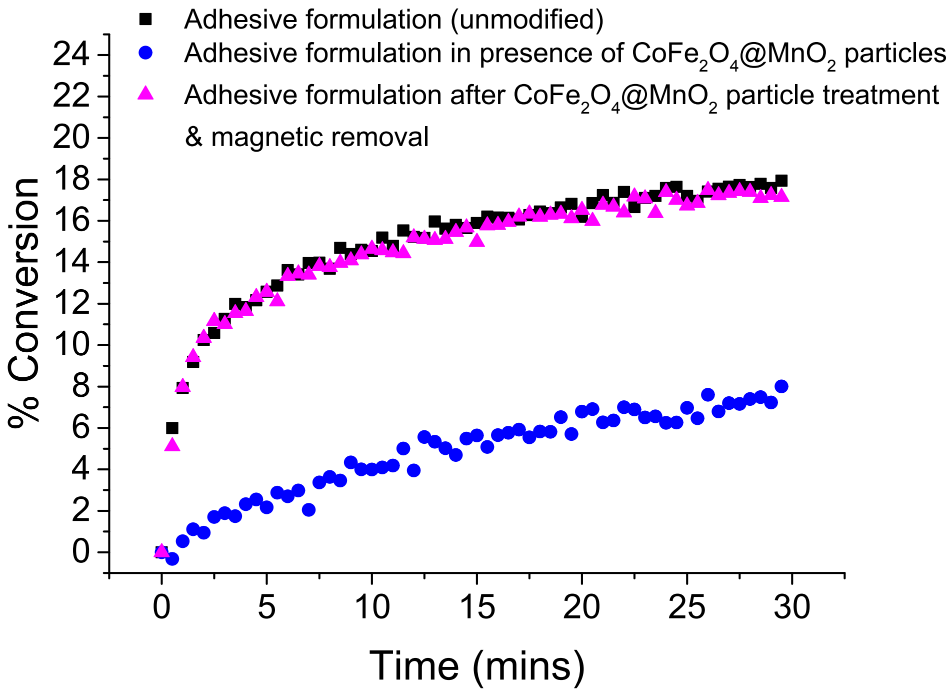

Figure S11. Plot of conversion of monomer (calculated from monitoring FTIR 1637 cm^{-1} peak representative of the disappearance of the C=C bond of TRIEGMA and hence polymerisation) with respect to time for the adhesive formulation in contact with a steel substrate. Samples measured: unmodified adhesive formulation (black), formulation with optimised $CoFe_2O_4@MnO_2$ nanoparticles present (blue) and after they had been removed (magenta).

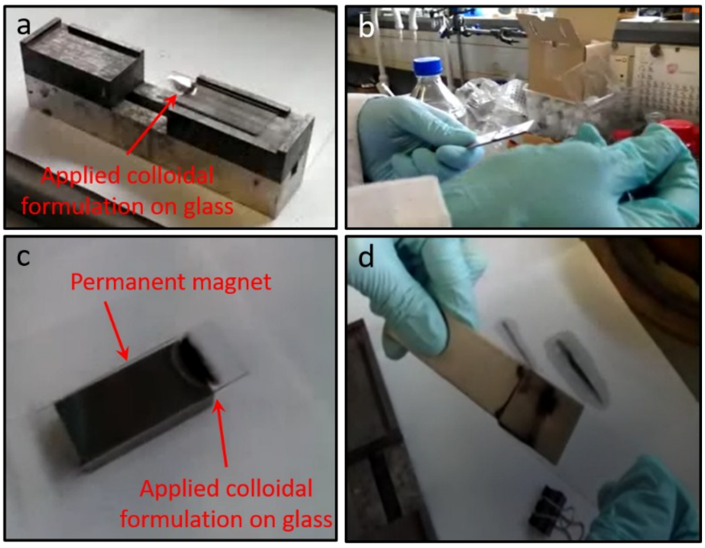

Figure S12. Still images showing glass-stainless steel plate combination adhesive tests: a) application of complete colloidal adhesive formulation containing $CoFe_2O_4@MnO_2$ particles to a glass plate; b) lack of plate adhesion in absence of a magnetic field; c) application of a permanent magnetic field (magnet indicated by arrow) to the joint with complete colloidal adhesive formulation applied, as per a); d) successful glass-stainless steel plate adhesion as a result of magnet application to the joint for 30 s.

Tables

Table S1. Ratios of MnO_2 and $CoFe_2O_4@MnO_2$ samples tested for adhesion inhibition properties and their adhesion capabilities (✓ for successful adhesion, ✗ for unsuccessful adhesion).

Sample	Mass ratio of particles:Cu^{II}	Deactivation of adhesion in presence of particles[a]	Magnetic removal[b]	Adhesion post magnetic removal[c]
MnO_2	1:1	✗	✗	-[d]
	2:1	✓	✗	-[d]
	3:1	✓	✗	-[d]
$CoFe_2O_4@MnO_2$	4.5:1	✓	✓	✗
	4:1	✓	✓	✗
	3.5:1	✓	✓	✓
	3:1	✗	✓	✓
	1.3:1	✗	✓	✓
	1:1.3	✗	✓	✓

[a] Deactivation of adhesion defined as prevention of reduction of Cu^{II} to Cu^{I}, polymerisation and hence no adhesion of metal plates using adhesion tests (Figure S1 and Experimental). [b] Ability to remove the nanocomposite sample system using an external magnetic field. [c] After removal of nanoparticles by magnetic field, assessment of supernatant adhesion as per [a]. [d] Not applicable due to lack of magnetic characteristics.

Table S2. Summary of the conditions required to achieve polymerisation (and hence metal substrate adhesion) in the absence and presence of nanoparticles (optimised ratios) and control systems (✓ for successful adhesion, × for unsuccessful adhesion).

Sample	Deactivation of adhesion[a]	Magnetic removal[b]	Adhesion post magnetic removal[c]
MnO_2[e]	✓	×	-[d]
Oleic acid[f]	×	×	-[d]
$KMnO_4$[g]	×	×	-[d]
$CoFe_2O_4$[h]	×	✓	✓
$CoFe_2O_4@MnO_2$[i]	✓	✓	✓

[a] Deactivation of adhesion defined as prevention of reduction of Cu^{II} to Cu^{I}, polymerisation and hence no adhesion of metal plates using adhesion tests. [b] Ability to remove the sample system using an external magnetic field. [c] After removal of nanoparticles by magnetic field, assessment of adhesion as per [a]. [d] Not applicable due to lack of magnetic characteristics. [e] 2:1 mass ratio of particles:Cu^{II}. [f] 5:1 mass ratio of oleic acid:Cu^{II}. [g] 1:1 mass ratio of $KMnO_4$:Cu^{II}. [h] 5:1 mass ratio of particles:Cu^{II}. [i] 3.5:1 mass ratio of particles:Cu^{II}.

Video Files

Video files complement Figure 6, main manuscript.

Video S1. Real time video of complete colloidal formulation applied between two aluminium plates in the absence of a permanent magnetic field showing no adhesion takes place.

Video S2. Real time video of complete colloidal formulation applied between two aluminium plates in the presence of a permanent magnetic field, for 30 s, showing successful adhesion takes place.

References

1. G.-L. Davies, S. A. Corr, C. J. Meledandri, L. Briode, D. F. Brougham and Y. K. Gun'ko, *ChemPhysChem*, 2011, **12**, 772-776.
2. C. M. Julien, M. Massot and C. Poinsignon, *Spectrochimica Acta Part A: Molecular and Biomolecular Spectroscopy*, 2004, **60**, 689-700.
3. M. V. Limaye, S. B. Singh, S. K. Date, D. Kothari, V. R. Reddy, A. Gupta, V. Sathe, R. J. Choudhary and S. K. Kulkarni, *The Journal of Physical Chemistry B*, 2009, **113**, 9070-9076.
